# Advances in Diagnosis of Skin and Superficial Tissue Disorders—“Old and Emerging” Diagnostic Tools

**DOI:** 10.3390/diagnostics14212414

**Published:** 2024-10-30

**Authors:** Costantino Ricci

**Affiliations:** Pathology Unit, DIAP-Dipartimento InterAziendale di Anatomia Patologica di Bologna, Maggiore Hospital-AUSL Bologna, 40133 Bologna, Italy; costantino.ricci4@unibo.it

Skin and superficial tissue disorders (SSTDs) are some of the most common diseases affecting humans. SSTDs cover an incredibly wide range of different diseases, including the neoplastic, degenerative, inflammatory, autoimmune, and many others. They are associated with significant mortality, morbidity, and costs for healthcare systems. Early diagnosis of this group of diseases and their complications is crucial to reduce morbidity, mortality, healthcare costs, and associated conditions. In recent years, the rapid development of technological and computer tools has led to the emergence of a large number of innovative diagnostic techniques (“emerging” tools) that have literally revolutionized the diagnosis and treatment of SSTDs, and their relationship with established diagnostic techniques (“old” tools) needs to be clarified [1,2,3,4,5,6,7,8,9,10,11,12,13,14,15,16,17,18,19,20,21]. These innovative techniques can assist dermatologists in the “classical” examination of the patient and significantly improve diagnostic accuracy with considerable time savings [videodermoscopy, in vivo reflectance confocal microscopy, line-field confocal optical coherence (LC-OCT), etc.] [1,2,3,4,5,6]. On the other hand, some of these emerging diagnostic tools involve histologic and/or laboratory diagnosis (whole-exome sequencing, methylation and proteomic analysis, automated real-time PCR assay, and NGS), as well as the involvement of healthcare professionals not traditionally involved in the diagnosis of SSTDs, such as radiologists, bioengineers, and informatics specialists [high-resolution ultrasound imaging (HRUI), computational and deep learning-based systems, AI-based digital image analysis software] [7,8,9,10,11,12,13,14,15,16,17,18,19,20,21]. As the description of all these diagnostic tools is beyond the scope of this manuscript, we have focused only on the subjects of this Special Issue. Reflectance confocal microscopy (RCM) of the skin is an in vivo skin imaging technique that allows the study of several skin tumors and inflammatory conditions [1,2,3]. RCM uses a low-power laser beam to produce a horizontal and real-time view of the skin at a cellular-level resolution [1,2,3]. The different reflection index of cellular structures hit by the laser beam is captured and transformed by software into a two-dimensional grayscale image [1,2,3]. LC-OCT is a novel device able to reproduce a “virtual biopsy” of the skin by integrating the properties of reflectance confocal microscopy (cellular resolution) and optical coherence tomography (depth acquisition) [1,4,5,6]. LC-OCT has been developed over the last few years and was approved as a medical device in Europe in 2020 [1,4,5,6]. Since then, an impressive number of papers have been published on this technique, reflecting the potential application of LC-OCT in almost all dermatologic diseases [1,4,5,6]. HRUI makes it possible to accurately visualize the skin and subcutis, with modern ultrasound equipment and high-frequency transducers enabling an in-depth lateral spatial resolution to differentiate several histological layers (epidermis, papillary and reticular dermis, subcutis, etc.) [7,8,9,10]. In addition, HRUI combined with power Doppler imaging accurately visualizes the dermal vascular plexuses, thus enhancing the diagnostic potential of this revolutionary technique and leading to the modern definition of sonohistology [7,8,9,10]. Molecular biology techniques have been part of the diagnostic, prognostic, therapeutic, genetic, andhereditary characterization of SSTDs for many years, but new and increasingly complex techniques (and often much more expensive equipment requiring specific skills and expertise) are becoming available every day (whole-exome sequencing, methylation and proteomic analysis, automated real-time PCR assay, and NGS) [11,12,13,14,15,16,17]. Obviously, a dissertation on these molecular biology tools would require specialized books and dedicated literature and is beyond the scope of this editorial [11,12,13,14,15,16,17]. However, it is essential that any professional approaching SSTDs becomes familiar with these tools and routinely collaborates with molecular biologists and geneticists [11,12,13,14,15,16,17]. All of medicine and science have recently been transformed by the development and diffusion of software-based and, ultimately, AI-based techniques, which have opened up scenarios that would have been unimaginable just a few years ago [18,19,20,21]. Every day, thousands of manuscripts, patents, and diagnostic and therapeutic tools are published and introduced into the marketing space, making it difficult to keep up with all the news [18,19,20,21]. In the upcoming months, the contribution of these software-based and AI-based techniques to diagnostic routines will probably be clarified [18,19,20,21].

In summary, multiple “emerging” tools are becoming available every day for the diagnosis of SSTDs, thus requiring integration with “old” tools and a close collaboration between an increasing number of professionals and skillsets.
